# (Di­methyl­phosphor­yl)methanaminium iodide–(di­methyl­phosphor­yl)methan­amine (1/1)

**DOI:** 10.1107/S1600536813019004

**Published:** 2013-07-13

**Authors:** Guido J. Reiss

**Affiliations:** a Institut für Anorganische Chemie und Strukturchemie, Lehrstuhl II: Material- und Strukturforschung, Heinrich-Heine-Universität Düsseldorf, Universitätsstrasse 1, D-40225 Düsseldorf, Germany

## Abstract

The asymmetric unit of the title structure, C_3_H_11_NOP^+^·I^−^·C_3_H_10_NOP, consists of one (di­methyl­phosphor­yl)methanamine (*dpma*) mol­ecule, one (di­methyl­phosphor­yl)methanaminium (*dpma*H) ion and one iodide counter-anion. In the crystal, medium–strong to weak N—H⋯O and N—H⋯N hydrogen bonds connect *dpma*H cations and *dpma* mol­ecules into strands along [001]. The iodide counter-anions form only very weak hydrogen bonds. The crystal used for the diffraction study was found to be an inversion twin with a ratio of 0.83 (2):0.17 (2). The title structure is isotypic to that of *dpma*H[ClO_4_]·*dpma* [Buhl *et al.* (2013 ▶). *Crystals* **3**, 350–362].

## Related literature

For transition metal complexes of the *dpma* ligand, see: Dodoff *et al.* (1990 ▶); Borisov *et al.* (1994 ▶); Trendafilova *et al.* (1997 ▶); Kochel (2009 ▶). For transition metal complexes of the cationic *dpma*H ligand, see: Reiss (2013*a* ▶,*b* ▶). For *dpma*H^+^ salts, see: Reiss & Jörgens (2012 ▶); Buhl *et al.* (2013 ▶); Lambertz *et al.* (2013 ▶). For the term tecton, see: Brunet *et al.* (1997 ▶). For the graph-set analysis method, see: Grell *et al.* (2002 ▶).
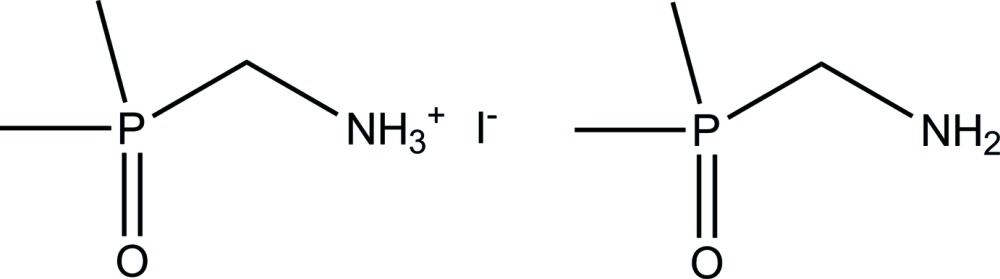


## Experimental

### 

#### Crystal data

C_3_H_11_NOP^+^·I^−^·C_3_H_10_NOP
*M* *_r_* = 342.09
Orthorhombic, 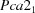

*a* = 17.7791 (3) Å
*b* = 11.1766 (2) Å
*c* = 6.91805 (12) Å
*V* = 1374.69 (4) Å^3^
*Z* = 4
Mo *K*α radiation
μ = 2.54 mm^−1^
*T* = 100 K
0.36 × 0.19 × 0.10 mm

#### Data collection

Oxford Diffraction Xcalibur Eos diffractometer
Absorption correction: analytical [*CrysAlis PRO* (Oxford Diffraction, 2009 ▶), based on expressions derived by Clark & Reid (1995 ▶)] *T*_min_ = 0.538, *T*_max_ = 0.808
10904 measured reflections
3062 independent reflections
2972 reflections with *I* > 2σ(*I*)
*R*_int_ = 0.023

#### Refinement

*R*[*F* ^2^ > 2σ(*F* ^2^)] = 0.016
*wR*(*F* ^2^) = 0.033
*S* = 1.05
3062 reflections
145 parameters
6 restraints
H atoms treated by a mixture of independent and constrained refinement
Δρ_max_ = 0.32 e Å^−3^
Δρ_min_ = −0.28 e Å^−3^
Absolute structure: Refined as an inversion twin.
Flack parameter: 0.173 (17)

### 

Data collection: *CrysAlis PRO* (Oxford Diffraction, 2009 ▶); cell refinement: *CrysAlis PRO*; data reduction: *CrysAlis PRO*; program(s) used to solve structure: *SHELXS97* (Sheldrick, 2008 ▶); program(s) used to refine structure: *SHELXL2013* (Sheldrick, 2008 ▶); molecular graphics: *DIAMOND* (Brandenburg, 2011) ▶; software used to prepare material for publication: *publCIF* (Westrip, 2010 ▶).

## Supplementary Material

Crystal structure: contains datablock(s) I, New_Global_Publ_Block. DOI: 10.1107/S1600536813019004/zl2559sup1.cif

Structure factors: contains datablock(s) I. DOI: 10.1107/S1600536813019004/zl2559Isup2.hkl

Supplementary material file. DOI: 10.1107/S1600536813019004/zl2559Isup3.cml

Additional supplementary materials:  crystallographic information; 3D view; checkCIF report

## Figures and Tables

**Table 1:** Hydrogen-bond geometry (Å, °)

*D*—H⋯*A*	*D*—H	H⋯*A*	*D*⋯*A*	*D*—H⋯*A*
N1—H11⋯N2^i^	0.86 (3)	1.95 (3)	2.805 (4)	173 (3)
N1—H12⋯O1^ii^	0.86 (3)	1.96 (3)	2.824 (3)	177 (4)
N1—H13⋯O2	0.91 (3)	1.81 (3)	2.716 (4)	172 (4)
N2—H21⋯O1	0.82 (3)	2.17 (3)	2.965 (4)	163 (4)
N2—H22⋯I1	0.83 (3)	3.21 (3)	3.948 (3)	150 (4)

Symmetry codes: (i) 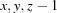; (ii) 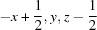.

**Fig. 1.**
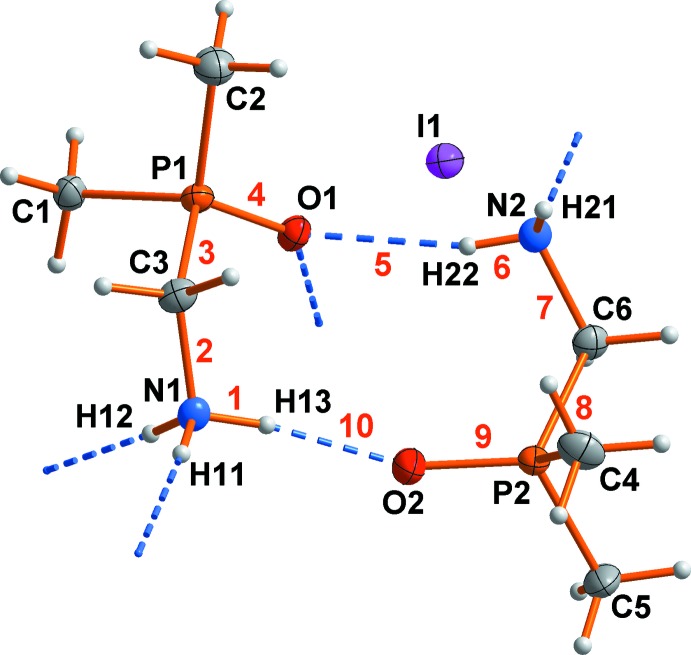
The asymmetric unit of the title structure is shown with displacement ellipsoids drawn at the 70% probability level. Blue broken lines indicate hydrogen bonds. The red number marks the ring size of the second level R22(10) graph-set descriptor for this connection of the dpmaH cation with the dpma molecule.

**Fig. 2.**
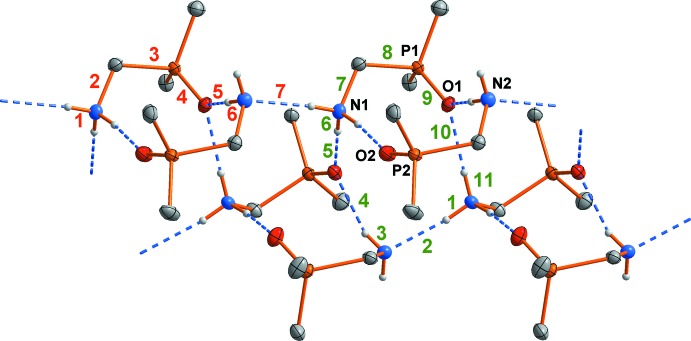
The primary units are further connected via hydrogen bonds to form strands running along [001].

**Fig. 3.**
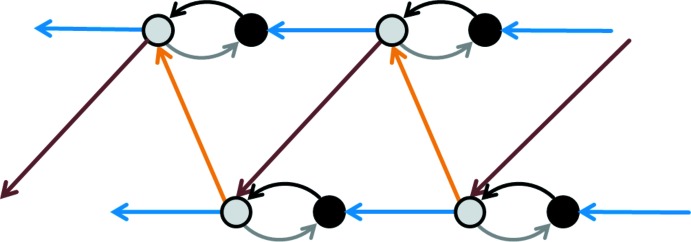
Constructor graph (Grell et al. 2002) of that part of the title structure shown in figure 2 (black dots: dpma, gray dots: dpmaH, arrows: crystallographic dependency is coded by the colours).

## supplementary crystallographic information

### Comment

(Dimethylphosphoryl)methanamine (*dpma*) is a promising bidentate ligand for the coordination of various transition metals (Dodoff *et al.*, 1990; Borisov *et al.* 1994; Trendafilova *et al.*, 1997; Kochel 2009). The corresponding N-protonated *dpma*H cation is also known to form transition metal complexes (Reiss, 2013*a*, 2013*b*). For simple *dpma*H salts it has been demonstrated that this tecton (for the term tecton see: Brunet *et al.*, 1997) shows a distinct tendency to form one-dimensional polymers by hydrogen bonded head to tail connections of adjacent cations (Reiss & Jörgens, 2012; Lambertz *et al.* 2013, Buhl *et al.* 2013). This contribution is part of our ongoing interest in the construction of new hydrogen bonded network architectures using phosphoryl containing tectons.

As illustrated in Figure 1, the asymmetric unit of the title structure consists of one *dpma*H cation, one *dpma* molecule, and one iodide anion in the non-centrosymmetric space group *Pca*2_1_. Bond lengths and angles within the *dpma*H cation and in the neutral *dpma* molecule are in the expected ranges (Buhl *et al.* 2013). The *dpma*H cation and the *dpma* molecule are each connected head to tail *via* one strong and one moderate N–H···O hydrogen bond constructing a ten-membered ring (second level graph-set descriptor: *R*^2^_2_(10); Figure 1 (red numbers)). Furthermore, these primary cyclic units are connected to adjacent units by N–H···N and N–H···O hydrogen bonds (Figure 2). These connections form one-dimensional strands running along [001]. The iodide counter anions form only very weak classical and non–classical hydrogen bonds, if at all. The hydrogen bonding motif of the backbone of the afore mentioned strands is represented by the second level graph-set descriptor C^2^_2_(7) (Figure 2, red numbers). The connections of the *dpma*H ^.^*dpma* cyclic units with adjacent ones produce one more simple ring-motif, which can be described as a third level graph-set descriptor: *R*^3^_4_(11) (Figure 2, green numbers). To visualize the basic principles of the construction a constructor graph is shown in Fig. 3 (Grell *et al.* 2002). The title structure is isotypic to the structure of *dpma*H[ClO_4_] ^.^*dpma* (Buhl *et al.* 2013). However, there are differences in detail due to the nature of the iodide counter anion. For *dpma*HI.*dpma* a 6% smaller volume of the unit cell has been determined. In both structures the counter anions are positioned in the vicinity of the amino group of the *dpma* molecule. The replacement of perchlorate by the significantly weaker hydrogen bonded iodide counter anion leads to a measurable strengthening of the hydrogen bond donating property of the amino group within the strands. The crystal which was used for the diffraction study was found to be an inversion twin with a ratio of 0.83 (2):0.17 (2).

### Experimental

In a typical experiment 0.5 g *dpma* was dissolved in 3 ml hydroiodic acid. The solution was slowly heated to dryness. The residual solid (*dpma*HI) and an equimolar amount of *dpma* were dissolved im 5 ml methanol. Slow evaporation of this solution at room temperature yielded colorless crystals of the title compound.

### Refinement

All hydrogen atoms were identified in difference syntheses. Hydrogen atoms at the methyl groups are idealized, were refined using rigid groups and allowed to rotate about the P—C bond (AFIX 137 option of the *SHELXL97* program; *U*_iso_ = 1.5*U*_eq_(C)). The hydrogen atoms at the methylene groups were included using a riding model with the *U*_iso_ values set to 1.2*U*_eq_(C). The coordinates of the hydrogen atoms involved in hydrogen bonds were refined with the N—H distance restrained to one common value. For each of these hydrogen atoms an individual *U*_iso_ value was refined.

### Crystal data

**Table:**

C_3_H_11_NOP^+^·I^−^·C_3_H_10_NOP	*D*_x_ = 1.653 Mg m^−^^3^
*M**_r_* = 342.09	Mo *K*α radiation, λ = 0.71073 Å
Orthorhombic, *P**c**a*2_1_	Cell parameters from 8874 reflections
*a* = 17.7791 (3) Å	θ = 2.9–29.4°
*b* = 11.1766 (2) Å	µ = 2.54 mm^−^^1^
*c* = 6.91805 (12) Å	*T* = 100 K
*V* = 1374.69 (4) Å^3^	Block, colorless
*Z* = 4	0.36 × 0.19 × 0.10 mm
*F*(000) = 680	

### Data collection

**Table:**

Oxford Diffraction Xcalibur Eos diffractometer	3062 independent reflections
Radiation source: Enhance (Mo) X-ray Source	2972 reflections with *I* > 2σ(*I*)
Graphite monochromator	*R*_int_ = 0.023
Detector resolution: 16.2711 pixels mm^-1^	θ_max_ = 27.5°, θ_min_ = 2.9°
ω scans	*h* = −21→22
Absorption correction: analytical [*CrysAlis PRO* (Oxford Diffraction, 2009), based on expressions derived by Clark & Reid (1995)]	*k* = −14→11
*T*_min_ = 0.538, *T*_max_ = 0.808	*l* = −8→8
10904 measured reflections	

### Refinement

**Table:**

Refinement on *F*^2^	Hydrogen site location: difference Fourier map
Least-squares matrix: full	H atoms treated by a mixture of independent and constrained refinement
*R*[*F*^2^ > 2σ(*F*^2^)] = 0.016	*w* = 1/[σ^2^(*F*_o_^2^) + (0.011*P*)^2^ + 0.370*P*] where *P* = (*F*_o_^2^ + 2*F*_c_^2^)/3
*wR*(*F*^2^) = 0.033	(Δ/σ)_max_ = 0.003
*S* = 1.05	Δρ_max_ = 0.32 e Å^−^^3^
3062 reflections	Δρ_min_ = −0.28 e Å^−^^3^
145 parameters	Extinction correction: *SHELXL2013* (Sheldrick, 2008), Fc^*^=kFc[1+0.001xFc^2^λ^3^/sin(2θ)]^-1/4^
6 restraints	Extinction coefficient: 0.00115 (12)
Primary atom site location: structure-invariant direct methods	Absolute structure: Refined as an inversion twin.
Secondary atom site location: difference Fourier map	Flack parameter: 0.173 (17)

### Special details

**Table:**

Experimental. CrysAlisPro, Version 1.171.34.44, (Oxford Diffraction, 2009). Analytical numeric absorption correction using a multifaceted crystal model based on expressions derived by Clark & Reid (1995).
Geometry. All e.s.d.'s (except the e.s.d. in the dihedral angle between two l.s. planes) are estimated using the full covariance matrix. The cell e.s.d.'s are taken into account individually in the estimation of e.s.d.'s in distances, angles and torsion angles; correlations between e.s.d.'s in cell parameters are only used when they are defined by crystal symmetry. An approximate (isotropic) treatment of cell e.s.d.'s is used for estimating e.s.d.'s involving l.s. planes.
Refinement. Refined as a 2-component inversion twin.

### Fractional atomic coordinates and isotropic or equivalent isotropic displacement parameters (Å^2^)

**Table:**

	*x*	*y*	*z*	*U*_iso_*/*U*_eq_	
I1	0.57385 (2)	0.73704 (2)	0.75007 (5)	0.01322 (6)	
P1	0.34627 (4)	0.54054 (8)	0.66655 (12)	0.00984 (17)	
O1	0.31186 (10)	0.62671 (19)	0.8072 (3)	0.0116 (5)	
N1	0.33299 (15)	0.7173 (3)	0.3886 (4)	0.0110 (6)	
H11	0.3537 (18)	0.748 (3)	0.288 (5)	0.025 (11)*	
H12	0.2881 (17)	0.693 (4)	0.364 (5)	0.033 (12)*	
H13	0.331 (2)	0.775 (3)	0.481 (6)	0.041 (13)*	
C1	0.28010 (15)	0.4322 (3)	0.5842 (4)	0.0125 (7)	
H1A	0.2643	0.3833	0.6909	0.019*	
H1B	0.3031	0.3827	0.4874	0.019*	
H1C	0.2372	0.4721	0.5299	0.019*	
C2	0.42584 (14)	0.4612 (2)	0.7564 (8)	0.0164 (6)	
H2A	0.4647	0.5170	0.7904	0.025*	
H2B	0.4442	0.4077	0.6586	0.025*	
H2C	0.4115	0.4161	0.8687	0.025*	
C3	0.38262 (16)	0.6192 (3)	0.4555 (5)	0.0124 (7)	
H3A	0.4316	0.6521	0.4865	0.015*	
H3B	0.3892	0.5624	0.3509	0.015*	
P2	0.35541 (4)	0.99276 (7)	0.78259 (13)	0.01040 (17)	
O2	0.32571 (12)	0.9051 (2)	0.6371 (3)	0.0154 (5)	
N2	0.38890 (15)	0.8119 (3)	1.0417 (4)	0.0120 (6)	
H21	0.370 (2)	0.771 (3)	0.957 (5)	0.016 (10)*	
H22	0.4349 (17)	0.809 (4)	1.022 (6)	0.033 (12)*	
C4	0.44922 (15)	1.0407 (3)	0.7318 (6)	0.0170 (7)	
H4A	0.4819	0.9723	0.7271	0.026*	
H4B	0.4659	1.0942	0.8315	0.026*	
H4C	0.4503	1.0811	0.6094	0.026*	
C5	0.30034 (16)	1.1262 (3)	0.7964 (5)	0.0179 (8)	
H5A	0.3036	1.1687	0.6761	0.027*	
H5B	0.3190	1.1760	0.8989	0.027*	
H5C	0.2488	1.1058	0.8217	0.027*	
C6	0.35496 (16)	0.9310 (3)	1.0246 (4)	0.0119 (6)	
H6A	0.3817	0.9854	1.1095	0.014*	
H6B	0.3033	0.9267	1.0695	0.014*	

### Atomic displacement parameters (Å^2^)

**Table:**

	*U*^11^	*U*^22^	*U*^33^	*U*^12^	*U*^13^	*U*^23^
I1	0.01355 (9)	0.01279 (10)	0.01334 (9)	0.00093 (6)	0.00029 (13)	0.00089 (17)
P1	0.0106 (4)	0.0092 (4)	0.0096 (4)	0.0003 (3)	0.0001 (3)	0.0014 (3)
O1	0.0137 (10)	0.0098 (12)	0.0112 (11)	−0.0002 (8)	0.0022 (8)	−0.0009 (8)
N1	0.0123 (14)	0.0099 (16)	0.0108 (13)	−0.0017 (11)	0.0011 (11)	0.0004 (12)
C1	0.0153 (15)	0.0105 (18)	0.0116 (15)	−0.0020 (12)	−0.0005 (13)	0.0001 (13)
C2	0.0149 (13)	0.0170 (15)	0.0172 (14)	0.0023 (11)	−0.0012 (17)	0.000 (3)
C3	0.0117 (15)	0.0118 (19)	0.0137 (15)	0.0010 (12)	0.0023 (13)	0.0011 (14)
P2	0.0105 (3)	0.0099 (4)	0.0108 (4)	0.0014 (3)	−0.0018 (3)	−0.0004 (3)
O2	0.0182 (11)	0.0133 (13)	0.0146 (12)	0.0035 (9)	−0.0032 (9)	−0.0035 (10)
N2	0.0126 (14)	0.0109 (16)	0.0125 (13)	−0.0015 (11)	−0.0009 (11)	0.0000 (13)
C4	0.0162 (14)	0.0208 (17)	0.0141 (19)	−0.0016 (11)	0.0015 (16)	0.0050 (19)
C5	0.0218 (15)	0.0124 (17)	0.020 (2)	0.0065 (12)	−0.0059 (13)	−0.0033 (13)
C6	0.0113 (15)	0.0113 (18)	0.0130 (15)	−0.0014 (12)	0.0006 (12)	0.0012 (13)

### Geometric parameters (Å, º)

**Table:**

P1—O1	1.500 (2)	P2—O2	1.501 (2)
P1—C2	1.782 (3)	P2—C4	1.787 (3)
P1—C1	1.782 (3)	P2—C5	1.787 (3)
P1—C3	1.823 (3)	P2—C6	1.811 (3)
N1—C3	1.481 (4)	N2—C6	1.467 (4)
N1—H11	0.86 (3)	N2—H21	0.82 (3)
N1—H12	0.86 (3)	N2—H22	0.83 (3)
N1—H13	0.91 (3)	C4—H4A	0.9600
C1—H1A	0.9600	C4—H4B	0.9600
C1—H1B	0.9600	C4—H4C	0.9600
C1—H1C	0.9600	C5—H5A	0.9600
C2—H2A	0.9600	C5—H5B	0.9600
C2—H2B	0.9600	C5—H5C	0.9600
C2—H2C	0.9600	C6—H6A	0.9700
C3—H3A	0.9700	C6—H6B	0.9700
C3—H3B	0.9700		
			
O1—P1—C2	114.65 (19)	H3A—C3—H3B	107.7
O1—P1—C1	112.00 (13)	O2—P2—C4	113.09 (16)
C2—P1—C1	107.30 (15)	O2—P2—C5	112.85 (13)
O1—P1—C3	110.77 (14)	C4—P2—C5	105.77 (15)
C2—P1—C3	103.78 (18)	O2—P2—C6	111.70 (14)
C1—P1—C3	107.81 (15)	C4—P2—C6	107.47 (17)
C3—N1—H11	107 (2)	C5—P2—C6	105.44 (15)
C3—N1—H12	112 (3)	C6—N2—H21	106 (3)
H11—N1—H12	111 (4)	C6—N2—H22	115 (3)
C3—N1—H13	109 (3)	H21—N2—H22	106 (4)
H11—N1—H13	108 (3)	P2—C4—H4A	109.5
H12—N1—H13	109 (4)	P2—C4—H4B	109.5
P1—C1—H1A	109.5	H4A—C4—H4B	109.5
P1—C1—H1B	109.5	P2—C4—H4C	109.5
H1A—C1—H1B	109.5	H4A—C4—H4C	109.5
P1—C1—H1C	109.5	H4B—C4—H4C	109.5
H1A—C1—H1C	109.5	P2—C5—H5A	109.5
H1B—C1—H1C	109.5	P2—C5—H5B	109.5
P1—C2—H2A	109.5	H5A—C5—H5B	109.5
P1—C2—H2B	109.5	P2—C5—H5C	109.5
H2A—C2—H2B	109.5	H5A—C5—H5C	109.5
P1—C2—H2C	109.5	H5B—C5—H5C	109.5
H2A—C2—H2C	109.5	N2—C6—P2	114.7 (2)
H2B—C2—H2C	109.5	N2—C6—H6A	108.6
N1—C3—P1	113.3 (2)	P2—C6—H6A	108.6
N1—C3—H3A	108.9	N2—C6—H6B	108.6
P1—C3—H3A	108.9	P2—C6—H6B	108.6
N1—C3—H3B	108.9	H6A—C6—H6B	107.6
P1—C3—H3B	108.9		
			
O1—P1—C3—N1	−40.2 (3)	O2—P2—C6—N2	49.4 (3)
C2—P1—C3—N1	−163.7 (2)	C4—P2—C6—N2	−75.2 (3)
C1—P1—C3—N1	82.7 (3)	C5—P2—C6—N2	172.3 (2)

### Hydrogen-bond geometry (Å, º)

**Table:**

*D*—H···*A*	*D*—H	H···*A*	*D*···*A*	*D*—H···*A*
N1—H11···N2^i^	0.86 (3)	1.95 (3)	2.805 (4)	173 (3)
N1—H12···O1^ii^	0.86 (3)	1.96 (3)	2.824 (3)	177 (4)
N1—H13···O2	0.91 (3)	1.81 (3)	2.716 (4)	172 (4)
N2—H21···O1	0.82 (3)	2.17 (3)	2.965 (4)	163 (4)
N2—H22···I1	0.83 (3)	3.21 (3)	3.948 (3)	150 (4)

Symmetry codes: (i) *x*, *y*, *z*−1; (ii) −*x*+1/2, *y*, *z*−1/2.
